# Early Diagnosis of Irkut Virus Infection Using Magnetic Bead-Based Serum Peptide Profiling by MALDI-TOF MS in a Mouse Model

**DOI:** 10.3390/ijms15045193

**Published:** 2014-03-25

**Authors:** Nan Li, Ye Liu, Zhuo Hao, Shoufeng Zhang, Rongliang Hu, Jiping Li

**Affiliations:** Military Veterinary Research Institute, Academy of Military Medical Sciences, 666 Liuying West Road, Jingyue Economic Development Zone, Changchun 130122, China; E-Mails: linan226@126.com (N.L.); liuye79@126.com (Y.L.); hao1107@126.com (Z.H.); zhangshoufeng@hotmail.com (S.Z.)

**Keywords:** lyssavirus, Irkut virus, rabies, early diagnosis, serum peptide profiling

## Abstract

Early diagnosis is important for the prompt post-exposure prophylaxis of lyssavirus infections. To diagnose Irkut virus (IRKV) infection during incubation in mice, a novel method using magnetic bead-based serum peptide profiling by matrix-assisted laser desorption/ionization time-of-flight mass spectrometry (MALDI-TOF MS) has been established. For this test, serum peptides were concentrated by adsorption to and elution from the magnetic bead-based weak cation ion exchanger. Mass spectrograms obtained by MALDI-TOF MS were analyzed using ClinProTools bioinformatics software. Construction of the diagnostic model was performed using serum samples from mice infected with IRKV and rabies virus (RABV) BD06, Flury-LEP, and SRV_9_ (as controls). The method accurately diagnosed sera 2, 4 and 8 days after IRKV and RABV infections. The sensitivity, specificity, and total accuracy of diagnosis were 86.7%, 95.2%, and 92.9%, respectively. However, IRKV could not be differentiated from RABV 1 day after infection. The results of the present study indicate that serum peptide profiling by MALDI-TOF MS is a promising technique for the early clinical diagnosis of lyssavirus infections and needs to be further tested in humans and farm animals.

## 1. Introduction

Rabies is an acute progressive encephalomyelitis. In general, it cannot be cured after the appearance of clinical symptoms, although it can be effectively prevented at the beginning of the incubation period by post-exposure prophylaxis [1]. More than 55,000 annual human deaths due to classical rabies virus (RABV) infection have been documented globally [2]. Furthermore, more than 99% of these fatal infections originate from infected carnivores in rabies-endemic countries such as India and China [3]. However, some human deaths may have been due to infections by other lyssaviruses, including Duvenhage virus, European bat lyssavirus types 1 and 2, Australian bat lyssavirus, and Irkut virus (IRKV), due to contact with infected bats. Indeed, bats efficiently transmit rabies or rabies-like infections to humans through shallow or intradermal bites, which often go unnoticed or are neglected. This leads to a lack of effective post-exposure treatment [4].

In 2012, an IRKV strain was isolated from a greater tube-nosed bat (*Murina leucogaster*) in Jilin Province, China [5]. A number of studies have shown that the structural proteins of this virus shared high sequence identity (more than 98%) with the IRKV isolate Ozernoe, Russia, which was obtained from an apparent human case of rabies after a bat bite [6,7]. Cross-reactivity with current rabies biologics was only partial [8,9]. Therefore, for the most effective prophylactic treatment, a system needs to be developed for the early detection of IRKV infection and for distinguishing IRKV infection from RABV infection in China.

Serum is easily accessible and can record different pathological conditions of patients; therefore, is one of the best sources for diagnosis of diseases. Serum peptide profiling can be used for the identification of novel biomarkers for the early detection of disease [10]. Matrix-assisted laser desorption/ionization time-of-flight mass spectrometry (MALDI-TOF MS) technology-based serum peptide spectra has many advantages, such as accurate and stable data, simple and quick operation, high-throughput, *etc.* [11]. Therefore, MALDI-TOF MS technology-based serum peptide spectra has been widely used for early diagnosis, pathogenesis interpretation, therapeutic targets selection in melanoma [11], breast cancer, and other solid tumors [12,13]. Recently, the technology has been employed for researching early detection of toxin and parasite infection [14,15]. However, there has been no report of detection of lyssavirus infection using MALDI-TOF MS technology-based serum peptide spectra. In the present study, we used this technique for the early detection of IRKV infection by distinguishing serum peptide profiling of IRKV- and RABV-infected mice.

## 2. Results and Discussion

After intramuscular injection with equal doses of IRKV-THChina12, BD06, and Flury-LEP, all adult mice presented with the clinical symptoms of rabies (lethargy, ruffled hair, muscle weakness, loss of body weight, and progressive paralysis of one or both hind limbs) between 6 and 13 days after infection, with death observed 24–48 h after the development of these symptoms. All of the SRV_9_-infected mice and the uninfected controls survived and remained healthy during the observation period. RABV BD06 represents street virus and RABV Flury-LEP and SRV_9_ represent attenuated viruses, with all of them having different virulence. They served as representatives of rabies virus strains for differentiation from IRKV infection. In the screening of magnetic beads, MB-WCX could enrich more serum peptides than other magnetic beads within the mass range of 1000 to 10,000 Da. (Figure S1 in supplementary data). Moreover, the smallest overlapping area was observed in the MB-WCX-pretreated serum samples (Figure S2 in supplementary data). The results of the present study indicate that MB-WCX was the optimum technique for the enrichment of serum proteins.

All mouse brain samples from the uninfected and SRV_9_-infected groups were negative by direct fluorescent antibody (DFA) test, and all mouse brain samples from BD06-, Flury-LEP- and IRKV-infected groups were DFA positive. Because the software analysis did not discriminate between serum samples collected 1 day after infection and those collected before infection (Figure S3 in supplementary data), the 30 serum samples collected 2 and 4 days after RABV BD06, Flury-LEP, and SRV_9_ infection were used as controls for the construction of a diagnostic model with the same amount of serum samples collected 2 and 4 days after IRKV infection. As shown in Table 1, the model could detect IRKV infection in mice serum at days 2, 4, 8 post-infection with high accuracy rates. The sensitivity, specificity, and total accuracy of diagnosis calculated using the four-fold table method were 86.7%, 95.2%, and 92.9%, respectively. Furthermore, a single sample was assayed in 20 min, *i.e*., from sample pretreatment to obtaining the result.

The DFA test is the gold standard method widely used for rabies diagnosis. However, the method is commonly used to detect brain samples for antemortem or postmortem diagnosis of rabies in animals. Currently, no method has been developed for early identification of lyssavirus infection. In this study, results suggested that the serum peptide profiling by MALDI-TOF MS could rapidly and accurately perform early diagnosis of IRKV infection in mice.

The diagnostic model for IRKV infection in mice detected 104 peaks in the mass range 1000–10,000 (*m*/*z*), five of which were statistically different between IRKV and RABV infections and could be regarded as potential biomarkers for diagnosis. Among the five peaks, two peaks with *m*/*z* ratios of 1746.07 and 4529.95 were upregulated, and the other three peaks with *m*/*z* ratios of 2806.67, 3951.39, and 4976.95 were downregulated in the IRKV-infected mice (Figure S4 in supplementary data). Because of the low concentration and purity of the potential biomarkers in serum samples, their amino acid sequences were not directly identified using MALDI-TOF MS. The separation and enrichment of the peaks are necessary to determine the origin and true nature of the potential biomarkers. In our previous study, the serum samples were subjected to high performance liquid chromatography and magnetic bead-based enrichment; however, sufficient concentration and purity of the targeted peptides were not achieved (data not shown). In brief, serum peptide profiling using MALDI-TOF MS can be easily performed, and therefore can provide additional insight into the development of novel diagnostic strategies.

## 3. Experimental Section

### 3.1. Viruses and Cells

IRKV IRKV-THChina12, which was obtained from a *M. leucogaster* bat in China [5], was amplified by a single intracerebral (i.c.) mouse passage. RABV BD06, which was isolated in 2006 from a rabid dog in China, was maintained in dog brains via serial passages. The RABV strain Flury-LEP, which is widely used as an attenuated vaccine for the prevention of dog rabies in China, was amplified by a single i.c. mouse passage. The RABV strain SRV_9_, which is an attenuated virus that causes death of suckling mice aged less than or equal to 13 days, was grown in BHK-21 cells. Virus titers (TCID_50_) were calculated by a method reported previously [16].

The BHK-21 cells were maintained in Dulbecco’s minimum essential medium supplemented with 2% newborn calf serum, 100 U/mL penicillin G, and 100 μg/mL streptomycin sulfate at 37 °C in a 5% CO_2_ humidified incubator.

### 3.2. Experimental Procedures

The Animal Welfare Committee of the Military Veterinary Research Institute, Changchun, China approved all of the animal experiments. Four-week-old SPF female adult BALB/c mice were obtained from the Changchun Institute of Biological Products (Changchun, China) and randomly divided into five groups of 30 mice each. Three groups were injected intramuscularly (i.m.) with 100 mouse LD_50_ (the lowest 100% i.m. lethal dose) of IRKV-THChina12, BD06, or Flury-LEP. The fourth group was injected i.m. with 100 TCID_50_ of SRV_9_, while the fifth group served as uninfected controls. Blood samples (approximately 50 μL) were drawn from the caudal vein of each mouse before infection and 1, 2, 4, 8 days after infection. Sera were separated by centrifugation at 5000× *g* for 10 min.

On the basis of the results of previous studies, the incubation period of rabies in mice was considered as 4 days after infection, and the clinical symptoms were considered to be presented 8 days after infection [5]. Mice that showed the clinical symptoms of rabies and those that survived 28 days of observation were euthanized by CO_2_ intoxication. Brains of the mice were removed and examined for the presence of RABV or IRKV antigens by the direct fluorescent antibody test [17].

### 3.3. Serum Pretreatment Using Magnetic Beads

For MALDI-TOF MS analysis, proteins and peptides from the serum samples were extracted by adsorption to and elution from magnetic beads as described previously [18]. Three different techniques of serum concentration using ClinProt™ microparticle beads (Bruker Daltonik GmbH, Bremen, Germany) were tested: magnetic bead–hydrophobic interaction chromatography (MB-HIC8, lot no. 11.219041.131001), magnetic bead weak cation ion exchange (MB-WCX, lot no. 11.223983.194001), and magnetic bead-immobilized metal affinity chromatography containing copper ions (MB-IMAC-Cu, lot no. 10.223364.115001).

### 3.4. Analysis of Serum Peptide Profiling

After calibration of an Autoflex III smartbeam-MALDI-TOF MS using the Bruker Protein Calibration Standard I Calibration kit after every 10 analyses, spectra from all serum samples were obtained under previously reported conditions using FlexControl (Bruker Daltonik) [18].

### 3.5. Construction and Validation of the Diagnostic Model

For the identification of biomarkers, normalization of the spectra obtained, internal signal alignment using prominent internal signal peaks, and a peak picking procedure using default settings were automatically performed using ClinProTools (version 2.2, Bruker Daltonik: Bremen, Germany). Visualization and statistical analyses were used to determine significant differences in the pretreated data generated by ClinProTools. The diagnostic models for IRKV infection were formulated using sera from RABV- (as control) and IRKV-infected mice by a genetic algorithm within the software suite, and the sensitivity, specificity, and total accuracy of early detection were calculated with SPSS 16.0 using the four-fold table method (SPSS Inc., Chicago, IL, USA) for Windows.

## 4. Conclusions

In the present study, an established diagnostic model was employed for the precise detection of IRKV infections in mice. The methods, based on serum peptide profiling, will be further established and evaluated for their potential in humans and farm animals as a novel clinical method for the early diagnosis of lyssavirus infections.

## Supplementary Information



## Figures and Tables

**Table 1.** Detection of IRKV infection using serum peptide profiling.

Group	No. of positive samples	No. of negative samples	Accuracy
Uninfected controls	7	113	94.2%
1 day after IRKV infection	17	13	56.7%
1 day after RABV infection	3	87	96.7%
2 days after IRKV infection	28	2	93.4%
2 days after RABV infection	3	87	96.7%
4 days after IRKV infection	29	1	96.7%
4 days after RABV infection	4	86	95.6%
8 days after IRKV infection	28	2	93.4%
8 days after RABV infection	6	84	93.3%
