# Supplementary material for: Early Diagnosis of Irkut Virus Infection Using Magnetic Bead-Based Serum Peptide Profiling by MALDI-TOF MS in a Mouse Model
Source: Int J Mol Sci. 2014 Mar 25;15(4):5193–8. doi: 10.3390/ijms15045193 (PMC4013557; doi:10.3390/ijms15045193)
Supplement: Supplementary file 1 [file ijms-15-05193-s001.pdf]

Supplementary Information

Figure S1. MALDI-TOF mass spectra of serum peptides from IRKV-infected mice that were measured using MB-HIC8, MB-WCX, and MB-IMAC-Cu.

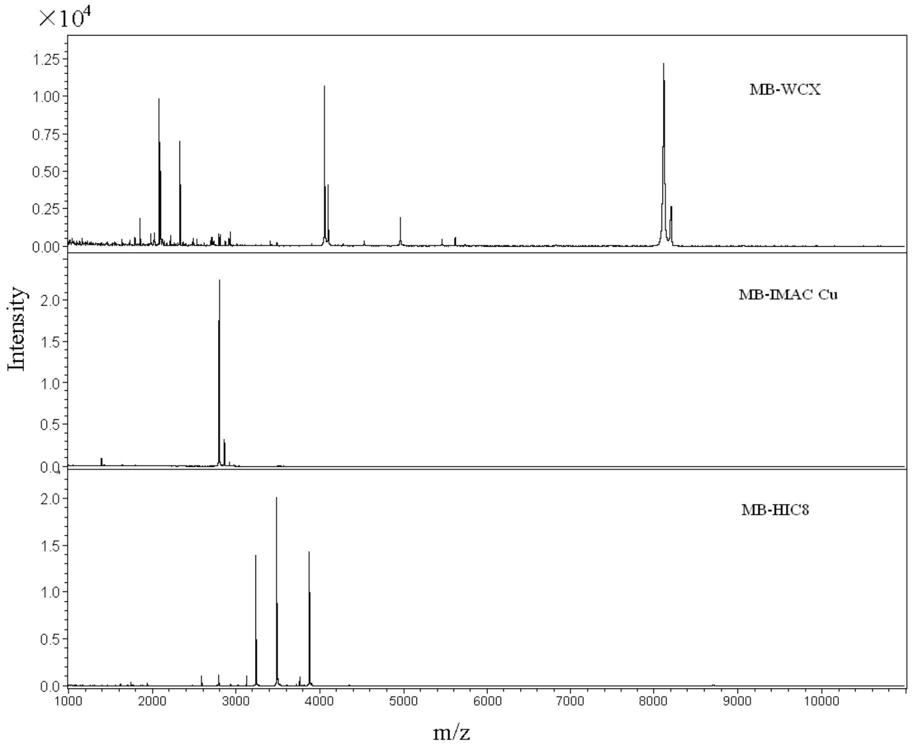

Figure S2. The distribution chart of serum samples from uninfected mice (x: uninfected control) and 8 days (○: IRKV infection) after IRKV infection. The coordinates represent the related protein intensities. The large ellipses represent the standard deviations of the peak area class average, and the small ellipses show that the protein peaks selected could distinguish IRKV infection from controls. (**A**) Sample distribution of MB-WCX-pretreated serum samples; (**B**) Sample distribution of MB-HIC8-pretreated serum samples; (**C**) Sample distribution of IMAC-Cu-pretreated serum samples.

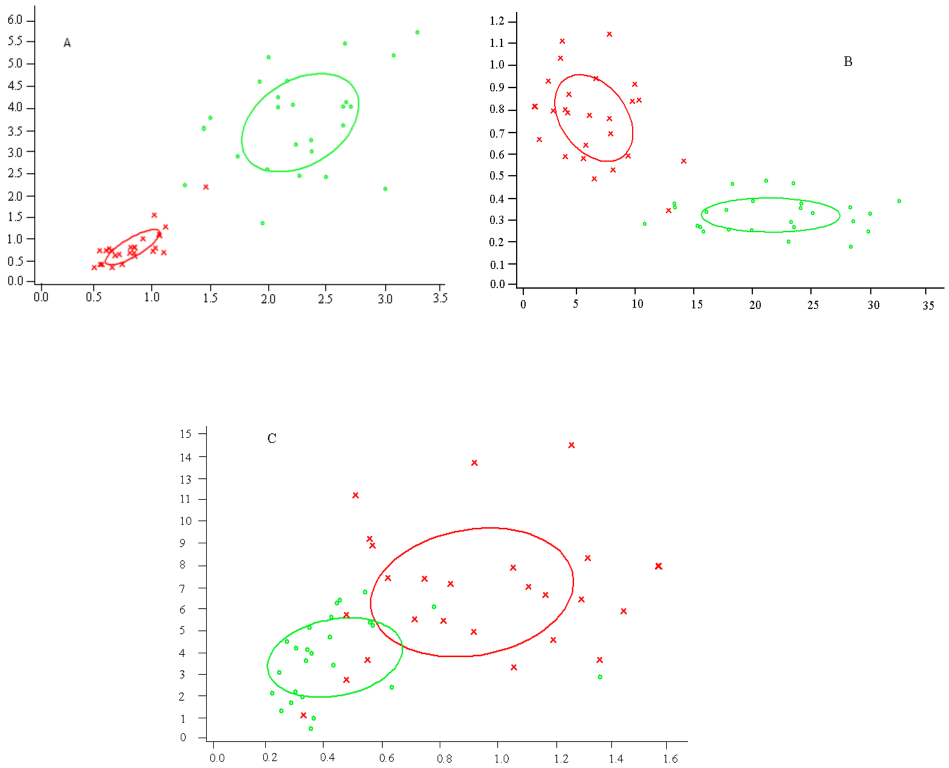

Figure S3. The distribution chart of serum samples from uninfected mice (x: uninfected control) and 1 day (○: IRKV infection) after IRKV infection. The coordinates represent the related protein intensities. The large ellipses represent the standard deviations of the peak area class average, and the small ellipses show that the protein peaks selected could distinguish IRKV infection from controls. There were overlapping areas between two groups, so this model could not discriminate serum from uninfected and 1 day IRKV-infected mice.

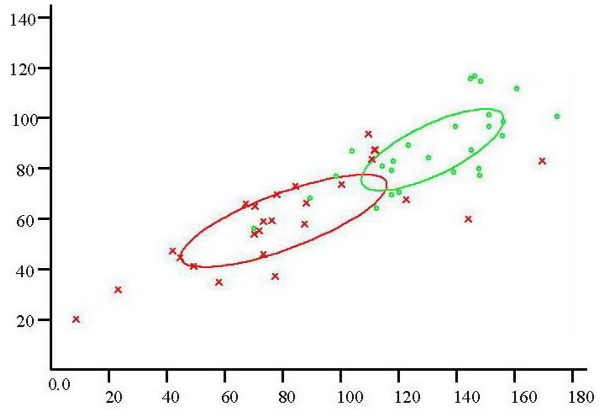

Figure S4. Different relative intensities of five peptide peaks between IRKV and RABV infections. (**A**) Upregulation of the peptide with MW of 1746.07 Da in IRKV infection group; (**B**) Upregulation of the peptide with MW of 4529.95 Da in IRKV infection group; (**C**) Down regulation of the peptide with MW of 2806.67 Da in IRKV infection group; (**D**) Down regulation of the peptide with MW of 3951.39 Da in IRKV infection group; (**E**) Down regulation of the peptide with MW of 4976.95 Da in IRKV infection group.

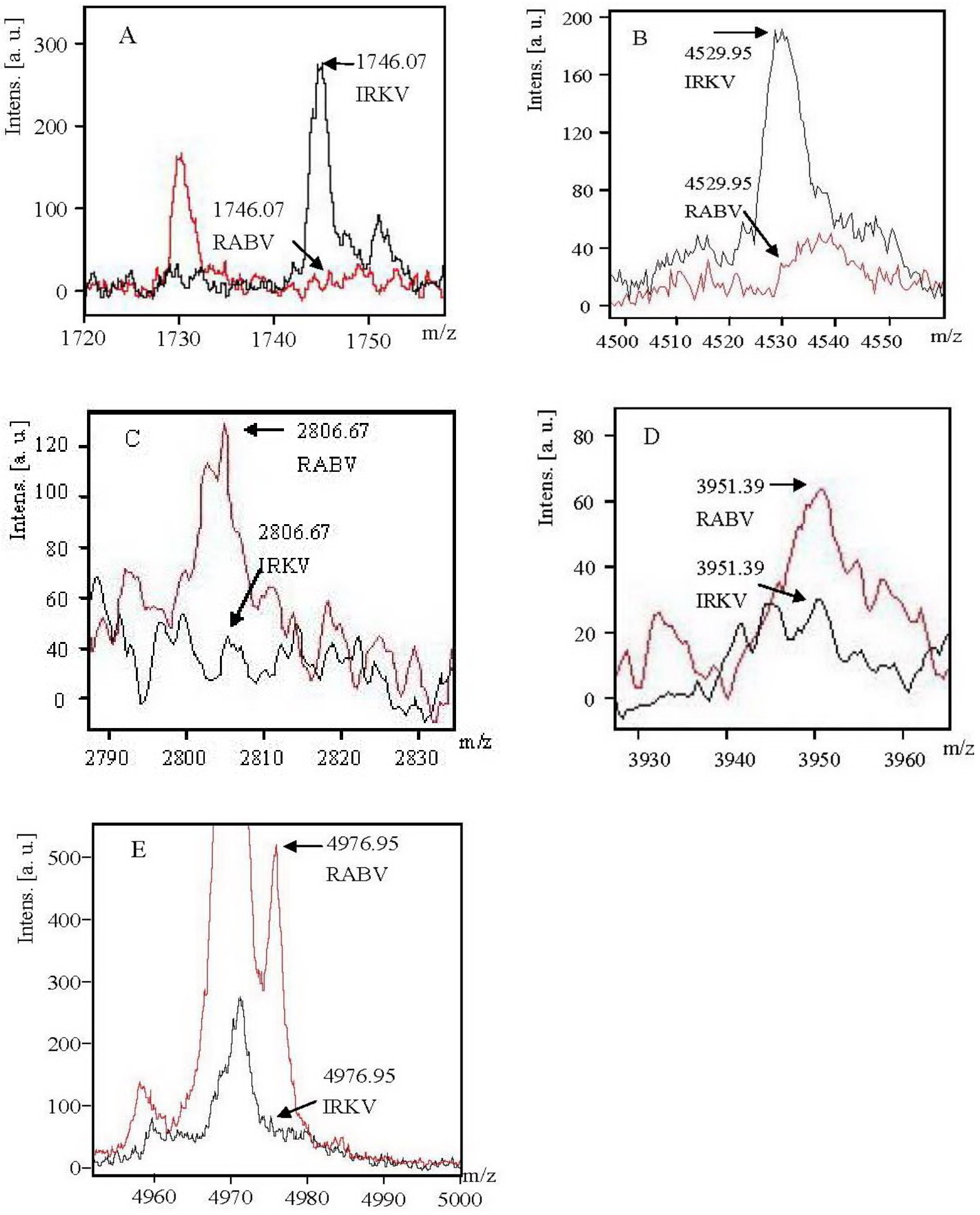

© 2014 by the authors; licensee MDPI, Basel, Switzerland. This article is an open access article distributed under the terms and conditions of the Creative Commons Attribution license (http://creativecommons.org/licenses/by/3.0/).
